# Biocompatibility and Clinical Application of Porous TiNi Alloys Made by Self-Propagating High-Temperature Synthesis (SHS)

**DOI:** 10.3390/ma12152405

**Published:** 2019-07-28

**Authors:** Yuri Yasenchuk, Ekaterina Marchenko, Victor Gunther, Andrey Radkevich, Oleg Kokorev, Sergey Gunther, Gulsharat Baigonakova, Valentina Hodorenko, Timofey Chekalkin, Ji-hoon Kang, Sabine Weiss, Aleksei Obrosov

**Affiliations:** 1 Research Institute of Medical Materials, Tomsk State University, Tomsk 634045, Russia; 2 Research Institute of Medical Problems of the North, Siberian Branch of the Russian Academy of Sciences, Krasnoyarsk 660017, Russia; 3 Kang and Park Medical Co., R&D Center, Ochang 28119, Korea; 4 Department of Physical Metallurgy and Materials Technology, Brandenburg University of Technology, 03044 Cottbus, Germany

**Keywords:** porous SHS TiNi, biocompatibility, rheological similarity, corrosion resistance, bone substitution

## Abstract

Porous TiNi alloys fabricated by self-propagating high-temperature synthesis (SHS) are biomaterials designed for medical application in substituting tissue lesions and they were clinically deployed more than 30 years ago. The SHS process, as a very fast and economically justified route of powder metallurgy, has distinctive features which impart special attributes to the resultant implant, facilitating its integration in terms of bio-mechanical/chemical compatibility. On the phenomenological level, the fact of high biocompatibility of porous SHS TiNi (PTN) material in vivo has been recognized and is not in dispute presently, but the rationale is somewhat disputable. The features of the SHS TiNi process led to a multifarious intermetallic Ti_4_Ni_2_(O,N,C)-based constituents in the amorphous-nanocrystalline superficial layer which entirely conceals the matrix and enhances the corrosion resistance of the unwrought alloy. In the current article, we briefly explore issues of the high biocompatibility level on which additional studies could be carried out, as well as recent progress and key fields of clinical application, yet allowing innovative solutions.

## 1. Introduction

Despite the fact that Nitinol was discovered in 1962 by William J. Buehler and further developed by Buehler and Frederick E. Wang in the U.S. Naval Ordnance Laboratory, its rheological similarity to biological tissues was reported for the first time in the 1980s [1,2]. Based on industrially deployed Nitinol, special TiNi-based alloys were developed, wherein the narrow temperature gap of austenite transformation was shifted towards a body temperature. This allowed shape memory implants made of these alloys to be congruent with biological tissues that are subjected to alternating physiological loads in the aggressive environment [3,4,5]. Whenever Nitinol is mentioned in the context of biomaterials or long-term implantable devices, a combination of corrosion resistance and biocompatibility with tissues is assumed, which is the pivotal characteristic of this alloy [6]. When considering the principles making Nitinol very attractive for clinical utilization, it is to be noted that it is economically justified regarding treatment cost minimization with a high performance.

There are a few general requirements concerning metallic materials clinically deployed. First, the material must have an appropriate viscoelastic potential, as regards the level of stress and frequency occurring in the corresponding part of the body. Secondly, it should possess a sufficient level of corrosion resistance, taking into account the implantation period and mechanical factors associated with the corrosion process. Thirdly, it has to demonstrate sufficient biological inertness, which is determined by negligible cytotoxicity, mutagenicity, carcinogenicity, immunogenicity, and thrombogenicity. As such, Nitinol combines all these properties and belongs to a group of biomaterials whose usage complies with the provisions of bioinertness, biocompatibility, and biomechanics [7,8,9].

Some early attempts at product development of medical Nitinol devices have been made by Nitinol specialists, who were not clinicians or primarily design focused. On the other hand, not enough designers and clinicians have yet received the insight and understanding of the Nitinol features necessary for scaling up the new implant systems. This was, in turn, crucially important for the success of clinical utilization. A set of orthopedic and traumatic devices for osteosynthesis was suggested, tested, and approved [2]. Further, material science engineers, in collaboration with the medical community, studied and exploited devices for surgical management of various lesions and injuries in midface, spinal and abdominal surgeries, oncology, urology, dentistry, and cryosurgery [10,11,12].

In the 1980s, in the USSR (Siberian Physical-Technical Institute), porous TiNi alloys were obtained using the self-propagating high-temperature synthesis (SHS) process in an inert atmosphere, followed by successful clinical use of implant systems made of porous SHS TiNi [13,14]. The SHS method to synthesize refractory ceramic compounds was initially proposed and comprehensively described by Merzhanov et al. [15,16,17]. SHS, as a powder metallurgy method, turned out to be the most appropriate for the fabrication of the porous TiNi body having the specified characteristics [18,19]. Additionally, SHS is a versatile method that produces a variety of intermetallic compounds for various application tasks [17]. 

Recently, porous SHS TiNi (PTN) compounds have been reported [20,21] to have some features which significantly distinguish PTNs from porous materials obtained by other methods of powder metallurgy using the same reactants [22,23,24]. It happens that the porous body formation during the SHS reaction is accompanied with the genesis of nonmetallics (titanites, spinels, perovskites, glass-ceramics, etc.) and nanocrystalline, amorphous superficial layers concealing the pore walls, which are of great interest for the academic community and for clinical application. It highlights the further need to investigate the surface structure of PTNs used as bone substitutes and scaffolds for cell-tissue engineering. In fact, the surface layers of PTN serve as a protective barrier in the chlorine corrosive-active environment, including for biological fluids [25,26,27,28].

PTN exhibits martensite transformations (MT), showing the shape memory effect and superelastic behavior, which, however, are not pronounced as in Nitinol [29,30]. The known scientific complexity is due to the multiphase state of PTN. In the case of variable cyclic load applied to the PTN graft incorporated in the body, the full cycle (direct-reverse-direct) of MT repeatedly occurs in a corrosive environment. The rheological similarity to biological tissues coupled with the enhanced corrosion resistance of unwrought PTN is supposed to impart additional benefits to this material, making it a promising alternative to Ti-based alloys, whose nontreated surface may incur an adverse corrosion effect. Follow-up observations [31,32] evidenced the high adaptability level of PTN as a biomaterial striving to complement existing surgical techniques for improved patient tolerance. 

In the review, we discuss the main features of PTN alloys, of which advanced implants are made, in the context of improved biocompatibility, along with the key fields of clinical application where these implants were deployed.

## 2. Fabrication of Porous SHS TiNi

Almost all bone endografts made of high-porous TiNi alloys (porosity ≥ 60%) are fabricated by the SHS method. This porous body minimizes implants’ failures (stress-shielding effect) and, hence, the complication rates [33,34,35]. Although additive technologies merit close attention from the industrial community, the high-porous TiNi alloy fabricated by SHS has a number of advantages, even in comparison to other powder metallurgy methods, including sintering, hot isostatic press, spark plasma sintering, thermal explosion, etc., [36,37]. In SHS, the product is directly synthesized from Ti-Ni elemental reactants via the propagation of a combustion wave through a green powder compact. Once the synthesis is started, the heat of reaction sustains the reaction until all of the reactants have been consumed. With regards to the clinical application of PTN, it is crucial to preset the desired mechanical characteristics, the shape memory effect (SME), and the superelastic parameters at temperatures that suit the body tissues. The main physical-mechanical characteristics of PTNs are summarized in Table 1.

Briefly, to fabricate PTN, commercial powders of coarse titanium made by calcium hydride reduction (mean particle size of 80–100 µm) and carbonyl nickel (mean particle size of 10–15 µm) are mixed for a few hours in an air jar and vacuum-dried [38]. The green powder mixture is loaded in a quartz tube and then loose-compacted by tapping for 10 min to achieve a porosity of tapped green compacts of 60–65%. The charged quartz tube is then loaded into a reaction furnace under flowing argon gas with a heating rate of 10–15 °C/min and is ignited electrically. In the mode, SHS is considered to occur with the involved liquid phase in a narrow reaction zone, which propagates autocatalytically through the preheated green powder compact. The heating schedule and temperature profile are controlled with a thermocouple placed inside the green powder compact. Once the compound has been synthesized, the reactor is withdrawn and cooled in a water container.

The difference between SHS and reaction sintering lies in the kinetics of the heterogeneous reaction [14,39,40]. At the beginning, the exothermic reaction of SHS partially dissolves the green powder compact, followed by the liquid-phase reaction, which triggers and dictates the formation of the intermetallic constituents (TiNi, Ti_2_Ni, and TiNi_3_). The TiNi/Ti_2_Ni/TiNi_3_ ratio in the matrix may vary and depends on kinetic parameters of the heterogeneous reaction.

Impurities trapped in the reactants are also crucial in synthesizing the porous compound. In powder metallurgy, the use of high-purity reactants is encouraged as it affords the fabrication of homogeneous alloys exhibiting specific attributes. This concept is particularly accurate for sintered TiNi, whereas it is not reasonable for PTN. Vacuum sintering at constant degassing forces not all of the existing impurities in the reaction system to be thermally dissociated, wherein some are gasified and subsequently withdrawn. The remaining impurities form diverse phases, which deteriorate the matrix, affecting the performance of the resultant alloy. On the contrary, SHS is generally referred to as the layer-by-layer exothermic reaction mode in an argon flow atmosphere when the preheated powder compact is ignited at 250–500 °C. The incipience and evolution of intermetallic phases occur in a thin solid-liquid reaction layer in milliseconds. A sequential cycle begins and evolves in the reaction layer at 100–200 µm thick, which inherits the size and morphology of the previous reaction layer through the capillary spreading of the (Ti + Ti_2_Ni) eutectic liquid emanated from the reaction zone. 

Notably, in the layer-by-layer combustion mode, the following processes are revealed in the reaction layer: (i) Origin of the eutectic liquid in the contact area of Ti and Ni particulates; (ii) dissolution of particulates in the eutectic liquid, which catalyzes a drastic increase of the eutectic liquid; (iii) exothermic reaction between dissolved reagents and successive crystallization of TiNi_3_, TiNi, and Ti_2_Ni precursors from the liquid; and (iv) interdiffusion migration of Ni atoms into solid Ti particulates and Ti atoms into solid Ni particulates, followed by the formation of intermetallic constituents [21,23,39,41].

Since the solid-liquid reaction layer remains porous, thermally dissolved gas-prone impurities managed by reaction gases leave the over-pressured high-temperature reaction zone, filtering through the structuring zone. The latter, having less pressure, remains red-hot. At the same time, the reaction gases, having a distinct effect on the conductive-convective heat transfer mechanism, are evident as a heat-and-mass transfer principal agent. They capture a portion of the liquid and transfer it from the reaction zone towards the surface, forming voids in the structuring zone. Therefore, the matrix is rectified to a large extent, whereas the pore wall surfaces are concealed by the sophisticated shell. The given shell comprises amorphous-nanocrystalline phases of intermetallic oxycarbonitrides in the form of epitaxial strata (foamy onlay and dense bisubstrate), as reported in References [20,21]. Considering the chemical composition and structure, the shell can be classified as a cermet Ti_4_Ni_2_(O,N,C) layer [42,43,44]. In fact, such amorphous-nanocrystalline phases are implied to exhibit high corrosion resistance.

Thus, in contrast to sintering modulated by the scant liquid, when synthesis is lengthy and coincides throughout the entire powder compact by the solid-liquid phase transformation, SHS is the rapid process occurring in a similar way, but in the presence of the abundant liquid. Impurities trapped in the reactants do not dissociate and recombine entirely upon sintering, whereas those upon SHS are subjected to thermal dissociation and chemical decomposition, which further results in the formation of the amorphous-nanocrystalline superficial layer. It provides a greater tolerance against corrosion and does not hinder the viscoelastic behavior of cyclically loaded PTN. 

## 3. Characteristics of Porous SHS TiNi

The high in vivo/vitro inertness of PTN is conditioned by the negligible anodic dissolution of the dynamically loaded PTN sample in simulated body fluids [6,7,8,9,25,28]. In the early in vivo terms, the anodic passivity of the PTN scaffold is beneficial for the attachment, cytocompatibility, and proliferation of seeded precursor cells as it sustains the formation of manifold tissular variants, reported in References [45,46,47,48,49]. Afterwards, a newly formed interface (e.g., bone regenerate) owes its vitality to the two factors as follows: (i) Continuing superficial anodic passivity of the PTN scaffold and (ii) minimum viscoelastic discrepancy between the surrounding bone tissue and the PTN matrix. The latter shows high elasticity without deterioration of the mechanical characteristics at applied loads and it physiologically redistributes stresses between adjacent bone fragments. Loads evoked by surrounding tissues can often exceed 6–8% relative strain, which exceeds the allowable values for most metal implants, destroying their protective surface films and ultimately leading to their destruction [50]. Deposited corrosion-resistant gradient coatings turn out not to remedy this challenge as they are usually nonelastic and have low fatigue strength [51,52]. The surface layer of the unwrought PTN alloy nevertheless withstands multicycle deformation and maintains its inherent integrity with the viscoelastic matrix [53].

### 3.1. Structure and Phase Composition of the PTN Surface

**Figure 1:**
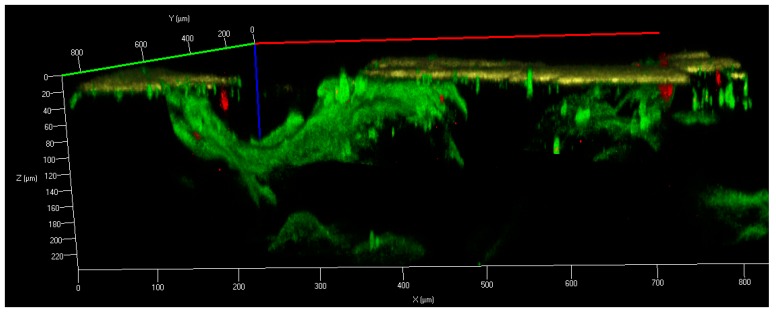
Confocal laser scanning micrograph of the thin-sectioned high-porous PTN specimen (wavelength—405 nm) [55].

Turning to the issue of the PTN enhanced corrosion resistance highlighted earlier, we were bound to note the superficial amorphous-nanocrystalline layer of intermetallic oxycarbonitrides, which entirely conceal the pore walls. It was denoted that the SHS process in itself is the rationale for this layer appearance resulting from retrograde gas streams interacting with the surface melt [43,54]. In our experience, we have studied the surface structures of high-porous PTN using a confocal laser scanning instrument [55]. The polished thin section is yellow, as can be seen in Figure 1, whereas the superficial layer, on which the focus was made, is represented as a translucent green film inside the unpolished open pore wall. It contains nonmetallic inclusions, observed as spatially distributed garnet flakes.

**Figure 2:**
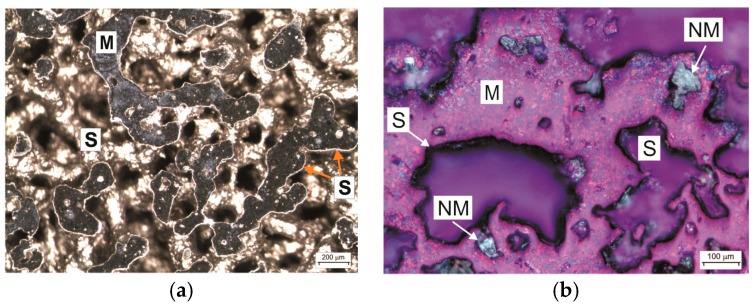
Light microscopy images of the thin-sectioned PTN in (**a**) a dark field with differential interference contrast (DIC) and (**b**) an ultraviolet polarized dark field [21].

The light microscope seemed to be a versatile instrument since it identified the massive superficial layer (S) concealing the sectioned matrix (M) in a dark field, as illustrated in Figure 2a [21]. Nonmetallic crystals (NM) can be distinguished in ultraviolet polarized light against both the matrix phase (M) and massive superficial layer (S), as depicted in Figure 2b.

**Figure 3:**
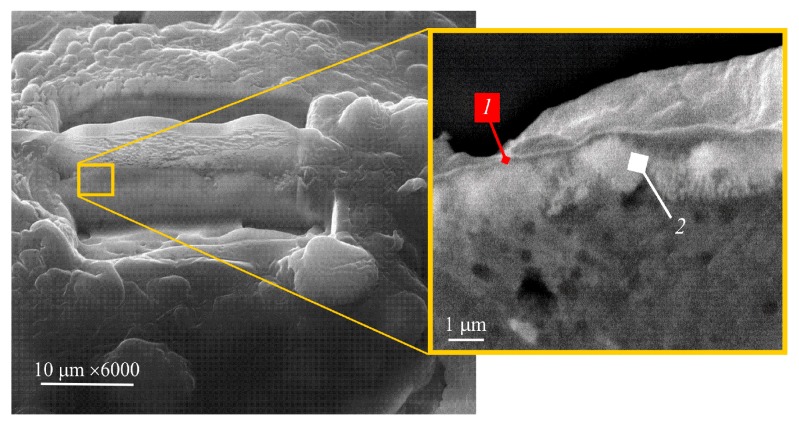
Structural features of the intricate sandwich (*1* + *2*), shown using a lamella taken from the open macropore wall of PTN (QUANTA 200 3D) [20].

**Figure 4:**
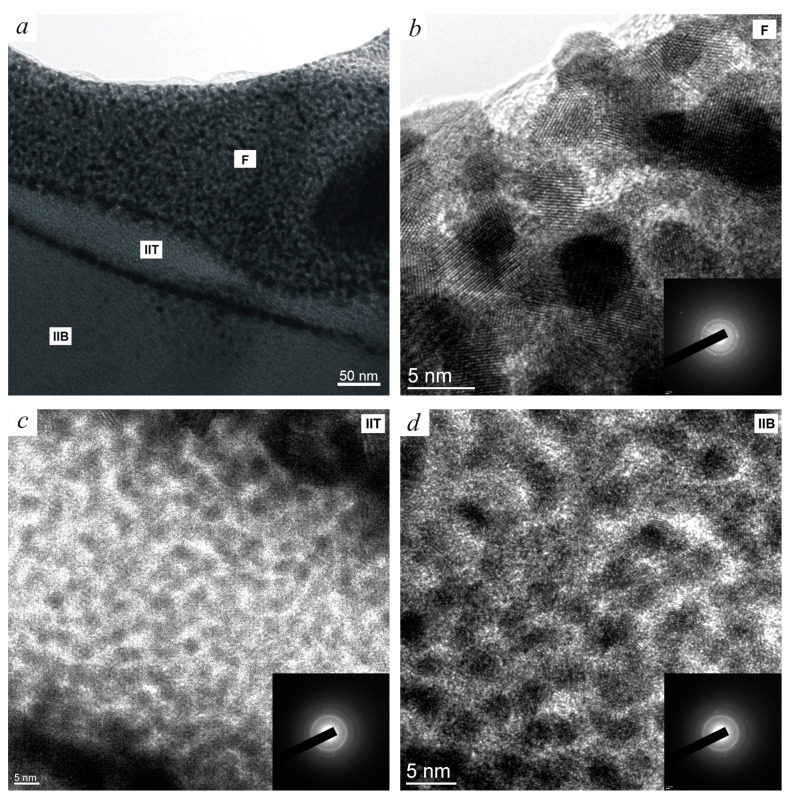
STEM images of lamella taken from the open macropore wall: (**a**) General view of the epitaxial layer [(IIB) + (IIT) + (F)] and individual structure of the (**b**) foamy onlay (F), (**c**) dense (IIT), and (**d**) (IIB) strata [21].

Furthermore, data emanated from the scanning tunneling electron microscope (STEM) and energy-dispersive X-ray spectroscopy (EDS) study of the (S) depicted in Figure 2 revealed its intricate structure (Figure 3). The foamy onlay (F) is seen to shell two dense sublayers (IIT + IIB), which are tightly bounded with the matrix (Figure 4). In Reference [20], it was previously reported that the (F) results from nanocrystalline intermetallic foam managed and dispersed by reaction gases, which is heterogeneous and discrete. High-resolution transmission electron microscopy (HREM), selected area electron diffraction (SAED), and EDS analyses allowed the authors to state that the presence of residual amorphous phases was obvious as well [21].

**Figure 5:**
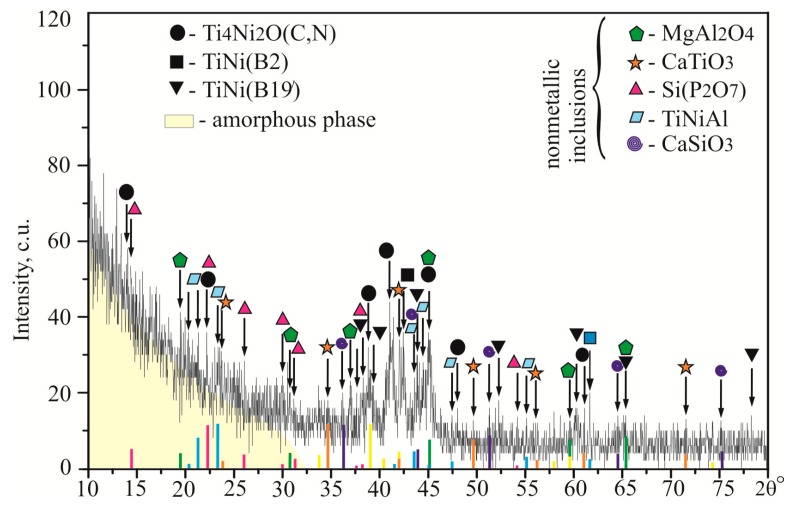
XRD pattern of a demolished PTN specimen, referred to the superficial layer [21].

Qualitative X-ray diffraction (XRD) analysis carried out on a demolished PTN sample to facilitate XRD-pattern acquisition of a non-uniform relief surface has strengthened the vision of the multifarious amorphous-nanocrystalline ensemble. The grazing beam at a low incident angle (<1°) was assumed to penetrate no more than a 100 nm in depth. XRD-patterns of the surface layers reported in Reference [21] were taken and brought together, as indicated in Figure 5.

The prevailing constituent, seen in Figure 5, is the amorphous-nanocrystalline phase of intermetallic oxycarbonitrides Ti_4_Ni_2_(O,N,C), whose fcc unit cell has been reported to appear due to oxygen, carbon, and nitrogen interstitial migration into the Ti_2_Ni lattice [56]. In fact, it can be considered as a solid solution of O, N, and C in the Ti_2_Ni phase. Reflexes belonging to the TiNi component appear in two crystallographic modifications of *B*2 (cubic parent) and *B*19’ (monoclinic martensite), simultaneously with Ti_4_Ni_2_O oxide. The crystallinity of the superficial layer at a depth of 100 nm is about 70%, 50–55% of which belongs to intermetallic oxycarbonitrides Ti_4_Ni_2_(O,N,C), whereas 10–15% goes to glass-ceramic and cermet phases, detected as CaTiO_3_, Si(P_2_O_7_), CaSiO_3_, MgAl_2_O_4_, and TiNiAl. The remaining 30% belong to the residual amorphous phases evident as a diffuse halo within 10–30°. 

Therefore, the discovered layers of amorphous-nanocrystalline Ti4Ni_2_(O,N,C), combined with glass-ceramic and cermet phases, impart corrosion-proof attributes expressed through electrochemical passivity in biological fluids, which is also consistent with Reference [57]. On the other hand, referring to the featured pore’s topography, it may be hypothesized that the foamy onlay (F) would maintain the promising bioactive characteristics, facilitating cell attachment and proliferation in vivo/vitro, as discussed below.

### 3.2. Rheological Resemblance of PTN to Biotissues

Most of PTN applications involve cyclically varying biomechanical loads that promote the need to fully understand the rheological behavior of this alloy. Although the accumulated knowledge on PTN can predict the post-implantation life-span of such implants, both in terms of stress-strain (total life) and damage tolerant (crack propagation) behavior, expanded information on their rheological characteristics needs to be highlighted. 

Viscoelastic rheological behavior of biological tissues is conditioned by their intricate structure [58,59]. In the 1950–1960s, collagen fibers were already considered as a key structure comprising all tissues, including bones [60,61]. Tropocollagen macromolecules mineralized with hydroxyapatite form a durable composite which is resistant against tensile and compressive loads. Moreover, the fact that bones are porous bodies underlines additional physical-mechanical features in their behavior. Bones containing tissue fluids do not fail over millions cycles of alternating load throughout their entire service life. With reference to Reference [62], the rationale for the remarkable functioning can be explained by the following factors. First of all, it is due to the cyclic viscoelastic characteristic of the organic matrix, in which collagen fibers are loaded, changing their conformation. Second, it owes to the elastic deformation of the mineral framework consisting of crystalline hydroxyapatite. Third, tissue fluids that fill the porous body of loaded bone redistribute the hydrostatic pressure through the bone so as to accommodate the severe strain to the level safe for collagen. As such, the porous bone structure, in which viscoelastic collagen fibers are mineralized by elastic hydroxyapatite crystals, is patterned on an anisotropic poroelastic composite material perceiving the external load. Viscous flows of tissue fluids infiltrating reciprocally through the osseous tissue contribute significantly to the viscoelastic rheological behavior of the bone, transmitting functional loads by means of fluid inertia and pressure gradients.

We can note the following arguments addressed to the provision of rheological similarity between PTN and bone tissue. 

(i) Regardless of which loading mode (axial, bending, or torsion) is applied, the minimum loads on the PTN bone graft from the host bone tissue cause linear (elastic) deformation of the PTN matrix, in which pore walls and interpore partitions undergo elastic cycling at a low strain magnitude (typically less than 2%). Higher loads are characterized by a nonlinear region, resulting in the onset of martensite transformation once a shear stress threshold of the PTN matrix is reached [63,64]. With that being the case, higher alternating dynamic loads, at constant temperature, appear to trigger the reversible austenite-martensite phase transformation, providing added value to the deformation process (up to 4%). Related to the general view of the deformation route, appearing structural defects, resulting from an increased deformation magnitude and transcended in localized areas of the PTN matrix, catalyze a crack formation and propagation, followed by the PTN bone graft or any part thereof eventually degrades until failure. Evidence in the literature indicates that the bone tissue behaves in a similar way [65,66]. The stress-strain behavior is characterized by a linear (elastic) region before a yield point, a post-yield nonlinear region containing the ultimate load, and a failure point at which the bone tissue can no longer carry the load.

(ii) The cyclic load applied to the PTN bone graft in vivo can be considered as a confluence of elastic and viscous deformation, which is due to the austenite-martensite transformation. This kind of combination is assumed to lead to stress relaxation and does not encourage the evolution of structural defects. The PTN rheological behavior within a viscous deformation region is consistent with that shown by the wet bone matrix ex vivo [67]. Of particular note is that the PTN body, having a certain pore size distribution, possesses a prominent capillary effect, which is sufficient to hold tissue fluids inside it [68]. This implies the PTN implant also has the possibility to transfer over most of the applied physiological loads via redistributed hydrostatic pressure, just as spongy bone tissue does. It must be acknowledged that a critical role, in this rheological context, is to be played by the ternary complex, consisting of the adjacent spongy bone, tissue fluid, and the poroelastic PTN implant. It acquires particular importance when substituting large defects of the loaded bones (e.g., femur, tibia, lumbar vertebrae). 

Numerous studies investigated the deformation behavior of porous TiNi compounds for the past ten years [29,63,69,70]. Most have reported that the task is fascinating and actual, but still challenging. As a rule, researchers carried out their tests in the axial loading mode. Although most of the studies were experimental, it was pointed out that compressed dry specimens exhibit viscoelastic deformation comparable to those mentioned above, but do not provide much information on a comprehensive understanding of the realities prevailing in vivo. To characterize the biomechanical interaction of the osteo-ligamentous interface with the engrafted PTN, a robust study of the PTN rheological patterns is believed to be needed, including tension, bending, and torsion tests performed ex vivo.

Definitely, viscoelastic PTN is rheologically different from a viscoplastic porous material. The latter, which possesses a high porosity, indicated an increased yield point (up to 6%), sustaining the irreversible viscous behavior of thin interpore partitions in compression testing [71]. PTN can be easily deformed by 4–6% [72,73], but the distinguishing peculiarity is that the noted strain is, for the most part, retained comparably to that shown by superelastic Nitinol [64,74]. The degradation behavior of PTN is strongly dependent on the phase-chemical composition, especially the brittle Ti_2_Ni phase network in the matrix as well as the intermetallic TiNi_3_ and nonmetallic phases responsible for the crack’s initiation, which is also true for dense TiNi.

### 3.3. Polarization Behaviour of PTN

Attempts have been made to protect the surface of Nitinol implants using various thin film technologies and, for the most part, they have made only modest progress [75,76,77,78,79]. Deposited coatings often suffer from microporosity, permeability, excessive thickness, and there is a mismatch in thermal expansion coefficients and elastic moduli between the substrate and coating. Therefore, coatings appear to be delamination-prone and have had little effect with regards to long-term protective films in vivo. These challenges are particularly relevant for porous alloys as the known methods using laser/electron-beam irradiation or sputtering are not effective. To some extent, this issue can be circumvented by using either plasma deposition or, occasionally, chemical deposition throughout the entire porous body as the most viable way of surface modification therein [80,81].

The osseointegration of bone substitutes can be facilitated by a change in the surface composition using bioactive ceramics. The short period of osseointegration of PTN bone graft may imply that its surface is chemically passive, bioactive, and cytocompatible, which can withstand the harsh conditions of medical applications. This could mean that the features of SHS have a positive effect on the chemical stability of the PTN surface, imparting chemical gradients inside the PTN implant to enhance cell viability. However, this matter remains poorly known and needs further study. We may address a peculiarity of the chemical resistance to the SHS process itself when nonmetallic impurities trapped in the reactants are thermally dissociated, followed by the surface of the synthesizing porous body which chemically absorbs them. Accordingly, the evolved superficial layer, having nanostructured attributes and high adhesion strength to the substrate, is tightly bounded with the matrix, concealing the latter.

**Figure 6:**
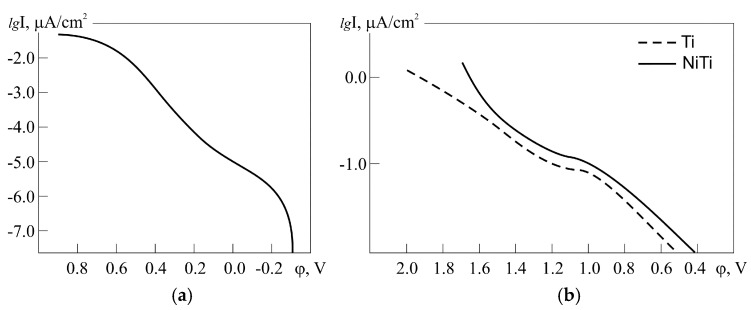
Anodic behavior of (**a**) unwrought PTN sample and (**b**) dense Ti and TiNi samples modified by anodic polishing and subsequent N ion implantation [43].

Potentiodynamic polarization was used in a comparative study of the surface susceptibility of PTN and dense Ti, TiNi, and in a 1% HCl solution, as reported in Reference [43]. Figure 6 illustrates the anodic behavior of unwrought PTN, dense Ti, and TiNi samples, modified by electropolishing, followed by N ion implantation. It should be noted that the findings shed additional light on the nature of unwrought PTN passivity. As seen, a corrosion measurement revealed that the anodic behavior of unwrought PTN in the chloride-containing environment mimics that of a modified TiNi sample in the passive region.

An early study on the electrochemical reaction has conversely shown that sintered porous Ti-based compounds undergo more corrosion [82]. Moreover, from the potentiodynamic polarization of porous TiNi fabricated by a powder metallurgy method using a high-purity ammonium bicarbonate powder and a blend of elemental reactants, it was reported that the studied sample was more susceptible to localized corrosion in a 0.9% aqueous NaCl solution as compared to the dense TiNi sample [83]. This also confirms our suggestion about the shielding superficial layer of intermetallic oxycarbonitrides, which entirely conceals the PTN rough surface, imparting a high corrosion resistance innate to cermets. The findings emanated from References [84,85,86], in which the authors explored the porous SHS TiNi alloy using a variety of instruments, accord well with our supposition. It is unlikely that the surface structure and the chemical composition of the studied samples are the same; however, we can inferentially assert that the PTN matrix is well protected by corrosion-proof strata consisting of intermetallic oxycarbonitrides and non-metallic inclusions.

## 4. Cytocompatibility of the PTN Surface

The spitted rough topography and biochemical aspect of the PTN surface play an important role in cell adhesion, growth, and proliferation, as a system of interconnected macro-/microvoids and grooves, which ultimately influence the biocompatibility of the hydrophilic PTN body in vitro/vivo [47,49,87,88]. Cell response to the surface topography is a primary feature of the forming tissue-specific variants. Surface roughness has a direct beneficial influence on cell morphology and proliferation. Moreover, the microporous surface structure can reduce a stress-shielding effect, encouraging the propitious tissue ingrowth [89]. On the contrary, the even surface prevents friendly cells adhesion and, in turn, decreases total biocompatibility. Literature confirmed that tissues ingrown on rough surfaces were stimulated towards differentiation [90,91], as shown by their gene expression in comparison with cells growing on smooth surfaces. For example, primary rat osteoblasts had higher proliferation, alkaline phosphatase, and osteocalcin expression on the rough surface compared to the smooth one [91]. Large size pores (>500 μm) may inhibit cell adhesion, reducing bone formation and vascular ingrowth. In contrast, small size pores (<100 μm) may hinder the diffusion of nutrients and metabolites but stimulate osteogenesis, reducing cell proliferation and forcing the implant’s incorporation. Therefore, the pore size distribution and average pore size of the PTN implant being developed are among the most important factors to strive towards for the right balance of the cell growth, proliferation, and tissue/vascular ingrowth herein [92].

The PTN scaffold has been reported to possess a wide range of pore sizes suitable for the cell cultivation in vitro, followed by the growth in vivo. Bone marrow cells (initial) seeded on the PTN scaffold attach to the pore walls, then actively grow and spread across the porous body. The cell growth and incipient intercellular substance cause reproduction of this cell mass, which contributes to colonization and subsequent differentiation [47,49]. The SEM control of cell attachment has indicated that the isolated initial cell tosses pseudopodia at a distance of 15 to 30 μm away by decoupling the chemotactic mechanism for the first 24 h. Further, solitary microfilament ends (less than 1 μm) were found to be attached and localized in superficial micropits of the pore wall. 

**Figure 7:**
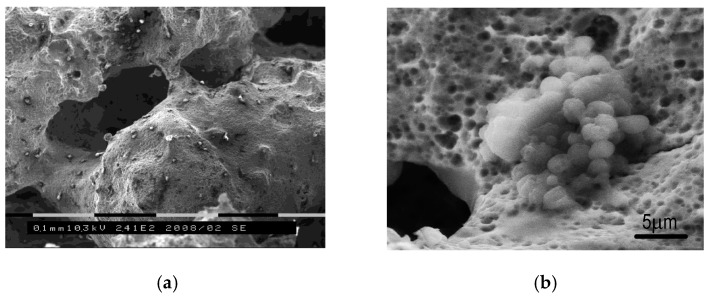
SEM image of (**a**) the cultured PTN scaffold in vivo and (**b**) micropits colonized by pancreas islet cells herein (on 7th day post-seeding) [47].

The SEM study of pore spaces was carried out in a 7 day time interval and the following features were noted: At the end of the first week, the cells were attaching and proliferated; most of the cells were fixed in local cavities, where there were many small pores less than 3 μm in size; and then, cells were actively growing (Figure 7).

The cell population continued to increase, as well as their growth in pores and the synthesis of the extracellular matrix. The 3D pore cluster allows them to proliferate intensively due to synthesis of the extracellular matrix and formation of spatial incrustations with different shapes and sizes (Figure 7a). 

**Figure 8:**
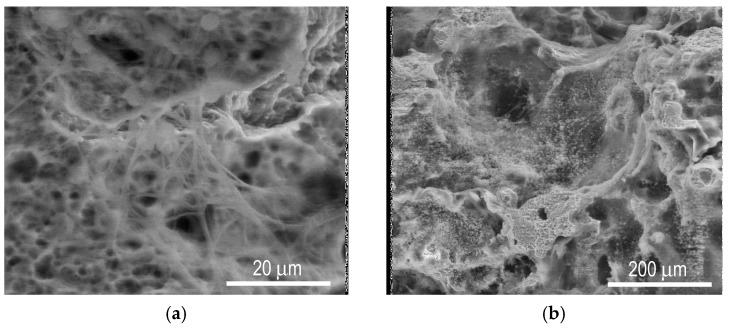

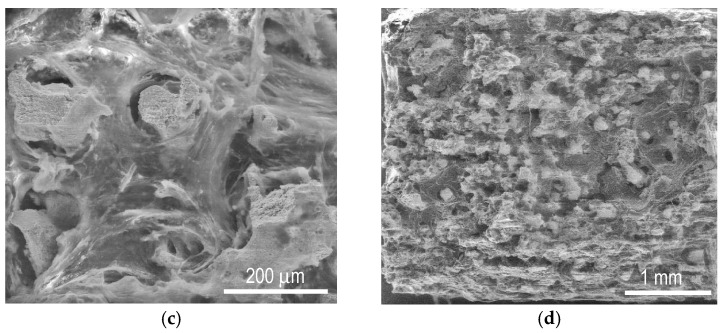
SEM images of cellular evolution in vivo: (**a**) Synthesized extracellular fibers and formation of spatial pseudopodia (on the 7th day); (**b**) gradual cellular ingrowth and formation of the extracellular matrix (on the 14th day); (**c**) phase of active infiltration (on the 21st day); and (**d**) PTN scaffold entirely filled by tissues (on the 28th day) [47].

Beginning on the 14th day, the tissue gradually lined the inner surface of pores and then the growth vector moved from the periphery towards the center, filling the entire pore space. During the fourth week, most of the pores were completely filled by cells and the differentiating matrix (Figure 8b). This effect was observed consistently from one week onwards, both in vitro (mesenchymal cells) and in vivo (hepatocytes and pancreas islet cells). The samples were ingrown by tissues and pores were filled by mesenchymal cells in 28 days and by hepatocytes and pancreatic islets in 40–60 days, respectively. 

In vitro experiments have shown that cells implanted into the PTN scaffold actively attach, grow, differentiate, and create a specific tissue structure even in vivo allogenic condition of the recipient.

Using various modes of the SHS process, it is possible to obtain different structures of the PTN scaffold with a specific pore size and distribution, which is very important in cellular and tissue engineering. With reference to References [93,94,95,96,97], a set of the mentioned features concerning the structure and characteristics of the PTN scaffold can be defined as follows:

- A well-developed spitted topography of pore walls (a large number of interconnected small pores and rough interpore partitions), which sustains the initial cell adhesion;
- Wetting ability, which facilitates saturation by water-soluble substances;
- Phase and chemical composition of the superficial strata, which has no inhibitory/toxic effect on cells seeded and growing tissular variants;
- Bio-mechanical behaviour, which is pretty similar to that exhibited by alive tissues.

The above-mentioned points are necessary for initial attachment, growth, and replication of host cells as a driving force for desired tissue to be harvested. Consistent cell growth and a short time whilst host cells colonize the PTN scaffold, competing with and conquering pathogenic cells, may emphasize the specific strengths for advanced bioengineering goals. The targeted differentiation of multipotential mesenchymal stromal cells of cartilage or osseous tissue inside the PTN scaffold proves the cytocompatibility of this material and extends the functional life-span of the incubated cells, prolonging the curative effect.

Thus, we can say that the PTN scaffold represents favorable conditions for the interactions of the host cell with the surface and expends the opportunities of bioengineering capabilities when considering morphological/functional properties of cells incubated herein. It can be used in a wide range of medical applications (management of locomotor apparatus diseases, metabolic disorders of the liver and pancreas, etc.). This material is designed to interact as long as it is in the body, in large deformations, exhibiting comparable rheological behavior and no graft vs. host response, which significantly distinguishes it from rivals.

## 5. Histological Studies of the Implanted PTN Scaffold in Vivo

To study the morphogenesis of reparative regeneration in the porous-permeable TiNi-based alloy, experimental studies were conducted on 10 mongrel dogs aged from 1 to 1.5 years, with weights ranging from 18 to 26 kg [98]. As-received PTN ingots were disintegrated into pellets. Bone defects were created in alveolar processes and then filled with porous granules. For the study of the microstructure characteristics, histological analysis of the material and the produced regenerate was performed at different times.

**Figure 9:**
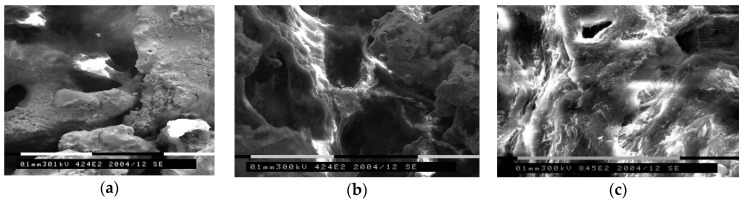
SEM images of tissue invasion through the PTN granules at (**a**) three, (**b**) seven, and (**c**) fifty-six days [98].

Figure 9 shows the filling of pores with tissue inside and between granules. New mature tissue is generated both on the surface and in pores, between PTN granules. On the first day of observation, islets of tissue start to form, mainly in large granules. On the 7th day, loose connective tissue can be observed between individual granules. In the course of time, the filling of the pores and the intergranular space with the newly formed multilayer tissue continues and it replicates the pore’s microrelief, which is in good agreement with the heterogeneous mechanism of bone formation. X-ray microanalysis of the tissues between granules and tissues that conceal the granules showed their similar composition [98]. The content of calcium, phosphorus, and potassium in the tissues corresponded to that in mature bone tissues. 

**Figure 10:**
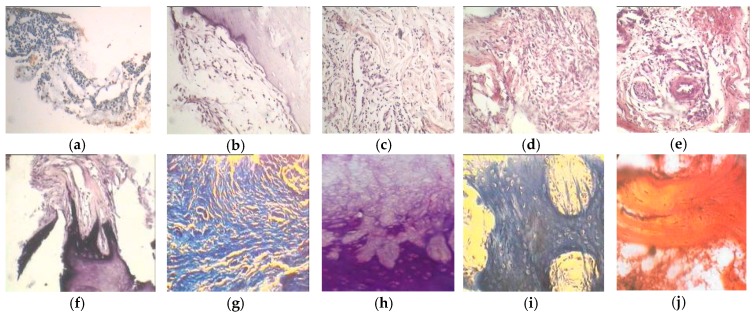
Dynamics of reparative processes in bone defects substituted by PTN granules at (**a**) three, (**b**) five, (**c**) seven, (**d**) ten, (**e**) fourteen, (**f**) seventeen, (**g**) twenty-one, (**h**) twenty-eight, (**i**) forty-two, and (**j**) fifty-six days [99].

Morphological findings from Reference [99] revealed that the cellular reticular tissue with sinusoidal capillaries, which comprises cellular elements of myeloid origin, is formed in the porous structure and between granules in a day (Figure 10). After two days, the number of leukocytes and fibroblasts in the generated tissue increased and thin fibrous structures appeared. By the 5th day, loose connective tissue was found closer to the defect edges, with fibers oriented along the bone trabeculae of the near-defect part of the bone. Separate cartilage cells were detected close to the defects in the lacunes. Further, the amount of cartilaginous elements decreased from the edges and the regenerate was a dense connective tissue with vessels that exhibited a muscular wall.

By the 7th day, the volume of the connective tissue component in the regenerate decreased since it was replaced with fibrous cartilage tissue. Closer to the defect center, dense loose connective tissue with collagen fibers was formed. After ten days, hyaline cartilage components were observed among the fibrous cartilage structures. During the following days, the replacement of connective tissue with fibrous cartilage tissue continued, which was then replaced with hyaline cartilage tissue. By the 42nd day, coarse fibrous bone tissue was detected on the defect edges and after 56 days, the regenerate mainly consisted of compact and spongy bone tissue. Further on, the resulting tissue did not noticeably change and was organotypic bone regenerate as an integral part of the implant material.

The fibrous cartilage is replaced by hyaline cartilage due to the formation of intercellular substances resulting from the chondroblastic activity, namely proteoglycans (e.g., chondroitin sulfates) supplanting the collagen fibers. In the recipient zone, the partial pressure of oxygen is known to increase due to the abundant periphery vascularization of the bone tissue, providing a rich blood supply to the margins. It makes pericytes, as a source of osteogenic cells, differentiate into osteoblasts under the effect of the high partial pressure of oxygen around them, whereas the function of osteoblasts is to form the intercellular substance of bone tissue. In so doing, active osteoblasts, modulating the intercellular substance of bone tissue, form the inorganic constituent known as osseomucoid. The latter, in turn, consists of calcium phosphate and hydroxyapatite crystals, which hinder the diffuse route of nutrients towards the hyaline cartilage. The scant diffuse nutrition leads to a situation in which the hyaline cartilage deteriorates and dies. Blood vessels with the same type of differentiation, controlled by pericytes, grow into the remaining space. This process ends when the hyaline cartilage is entirely replaced by coarse fibrous bone tissue, as seen in Figure 10 (depicting all stages of indirect osteogenesis).

Analysis of the reparative osteogenesis in bone defects after their reconstruction with PTN granules indicated that formation of bone regenerate through the ingrowth and differentiation of tissues occurred according to the patterns of indirect osteogenesis. At first, loose connective tissue forms, followed by the formation of dense irregular tissue, which results in the fibrous cartilage with signs of incipient hyaline cartilage. Further, the latter are gradually replaced by coarse fibrous bone tissue, transforming into mature spongy tissue.

## 6. Clinical Application of PTN Implants

### 6.1. Cervical Spine Superelastic PTN Cages

The specific characteristics of the anatomical structure of the vertebral bodies, the presence of shock-absorbing intervertebral discs, and special biomechanics of the vertebra exclude the use of traditional materials and structures in spinal surgery [100]. The properties and structure of the PTN cage for the vertebra are close to those of the spinal body, which ensures circulation of tissue fluids, plasma at the bone-implant boundary responsible for the metabolism of bone cells, and the formation of the bone-implant interface [9,28,45,86]. The contact surface resistant to aggressive biological fluids and the rheological behaviour of the PTN cage ensures its supportability, maintains the height of the vertebral body, and eliminates excessive loads without failure.

**Figure 11:**
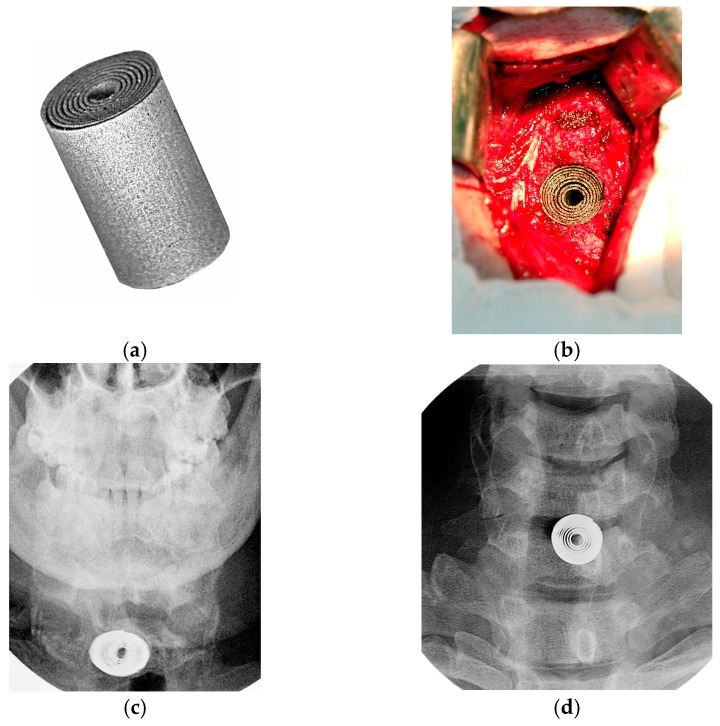
Cervical spine dynamic PTN cage: (**a**) General view; (**b**) intraoperative view of the surgical wound; X-ray images of the head tilted (**c**) down and (**d**) back in two years after surgery [101].

Forty-three patients suffering from cervical osteochondrosis received a dynamic PTN cage, whose shape is set as an eiloid cylinder, seen in Figure 11, for ventral interbody fixation of the cervical spine [12,32,101]. Since the PTN cage structure is superelastic, it is easy to attach the desired shape for insertion, followed by the implant deploys and self-locking in-situ, as depicted in Figure 11b. Due to the reliable elastic stabilization of the cervical spine, there was no need to wait when the bone block was formed, and all patients have been discharged the next day after surgery. The consistency of the operated vertebral motor segments allowed us to exclude postsurgical external immobilization of the neck. No complications associated with the implant features were noted in all cases. X-ray check performed 24 months after surgery indicated no evidence of migration, cracks, or failure of the implants. No areas of bone resorption were identified around the implants. Head flexion and extension radiographic images (Figure 11c,d) indicate preserved mobility in the cervical levels, which were managed using the PTN cage. Additionally, the range of motions in adjacent levels did not change, as can be seen in the Supplementary Video clip taken in the follow-up period.

All clinical cases indicated a high durability of the PTN cage structure with the maximum form change. In the follow-up period, the deformed PTN cage was noted to survive even in the case of high loads. When it is free from a load, the shape completely retains without any degradation. Notably, the phase transitions provoked by applied cyclic loads accommodate internal stresses throughout the PTN matrix and this is a justification for high performance under continuous cyclic loads.

### 6.2. Customized PTN Endografts in Maxillofacial Surgery

**Figure 12:**
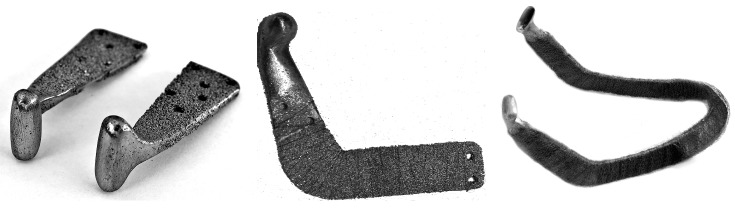
Customized PTN endografts for surgical repair of mandibular lesions [102].

For the first time, an experimental study on replacement of the mandible using a prototype of PTN was performed in 1982, whereas follow-up observation of this clinical case was reported in 1986 [2], and it is still feasible for clinical application. Customized combined endografts made of PTN and dense TiNi were developed to treat patients with mandibular lesions, including mandibular, maxillary and nasopharyngeal malignant tumors [102,103]. The PTN endografts of the mandibular ramus can be developed with right and left versions, including the head of the temporomandibular joint. The prosthesis consists of an ultra-elastic perforated plate, with porous parts of similar shape and size, fixed on both its sides. On the one hand, the structure exhibits a polished thickening that corresponds to the configuration of the head of the mandible (Figure 12). The size and configuration of the endoprosthesis are determined individually in accordance with computed tomography imaging and CAD modelling. Due to the superelasticity of the construct, it can be easily modified, depending on the shape required, to eliminate defects of the mental and lateral mandibular parts.

**Figure 13:**
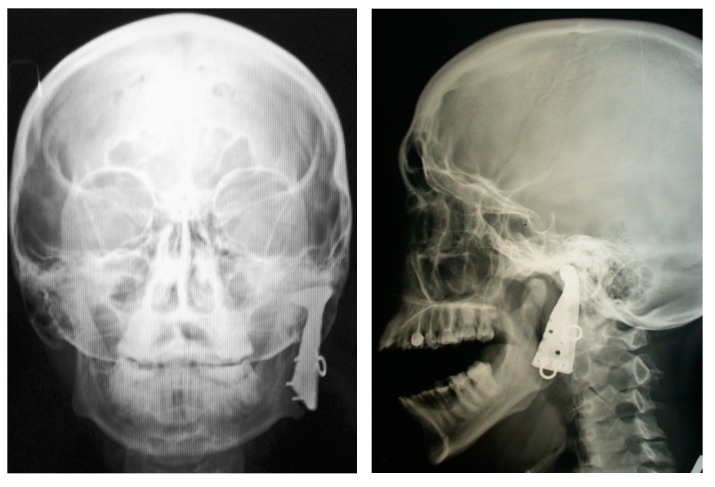
X-ray images of the repaired left mandible for deforming temporomandibular osteoarthrosis (a year after surgery) [102].

This ensures restoration of the anatomical architecture of the repaired area, normalizes the function of chewing and swallowing, and prevents secondary deformities caused by protruded bone fragments and scarring of soft tissues in the postoperative period, as illustrated in Figure 13.

Analysis of the follow-up observations in patients with destructive changes in the condylar processes proved the high efficiency of customized PTN endografts. Due to biochemical and biomechanical compatibility, combined endografts substituting tissue lesions behave congruently. Connective tissues from recipient areas ingrow the PTN body with negligible foreign body response and form an organotypic regenerate. The polished articular heads, from Figure 12, prevent adhesion with host tissues and maintain the necessary range of mandibular movements.

The use of customized PTN endografts for total and subtotal substitution of the mandible, including the condylar process and mandibular ramus, is thus a pretty good surgical method for reconstructing the anatomical features of the affected area.

### 6.3. Customized PTN Endografts in Oncosurgery

**Figure 14:**
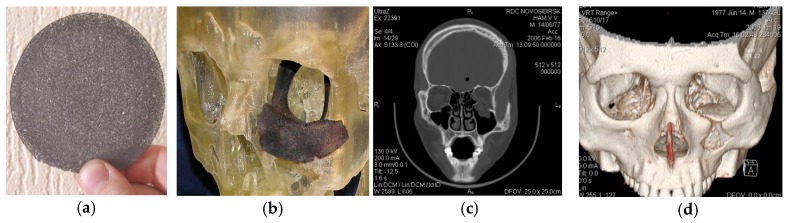
Repair of the orbit: (**a**) PTN disk plate before modeling, (**b**) preoperative customization using the 3D printed model; (**c**) coronal plane CT of the orbit before surgery; and (**d**) 3D reconstructed postsurgical CT of the repaired orbit [105].

Customized endografts made of PTN disk plates 0.3–0.4 mm thick, as depicted in Figure 14, were applied to replicate the maxilla, zygomatic bone, orbit, nose, and midface structures in cancer patients [104]. The superelastic property makes the implant flexible, which enables intraoperative modeling. The porous structure fixes the implant firmly in the wound, followed by connective and bone tissue ingrowth, which occurs with subsequent epithelialization of the postoperative cavity. The rigid central part and flexible edges of endoprostheses eliminate various discrepancies. The customized PTN graft can be produced based on CT scans and CAD modeled construct. At the same time, the implants provide good anatomical and aesthetic results in the elimination of complex defects on the walls of the orbit and its edges and adjacent bone structures (Figure 14). One of the adverse factors of midface reconstruction is highly virulent flora influencing the operating wound, which trigger the inflammation process in the implantation zone. Additionally, the subcranial region is an area of increased functional activity. It is clear that an endograft, in which resilience to the adverse impacts along with the anisotropic compliance and versatility in terms of stress-strain is inherent, can be the most advanced option. The superelastic feature of PTN is beneficial for smooth insertion through the minimal incision (the customized graft can be intraoperatively predeformed and shrunk for smooth insertion), followed by deploying within the orbital area in situ. This means that a concept of minimally invasive surgery is technically feasible. Moreover, in terms of stress-strain, the superelastic behaviour shown by PTN can counterbalance possible negative effects in the follow-up period. It is particularly important in pediatric patients or teenagers when the implanted PTN graft mimics the anisotropic compliance of the repaired orbit. So, in other words, the superelastic PTN as a load-bearing implant adapts to the augmenting midface/orbital environment, demonstrating higher adaptability without impairment of the mechanical performance at higher loads.

Clinical examples have shown that PTN grafts ensure reliable restoration of the inferior orbital wall, prevent displacement of the eyeball, correct vision errors, and eliminate undesirable aftereffects [105]. The properties of the PTN plate allow modeling of sophisticated implants at a certain temperature regime. The customized PTN grafts precisely render orbitozygomatic outlines and orbital floor, thus recovering the anatomical structure, and are supposed to be an attractive alternative to Ti-based plates.

### 6.4. PTN Implants in Traumatic Surgery

**Figure 15:**
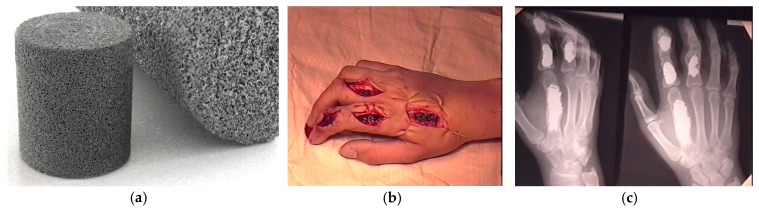

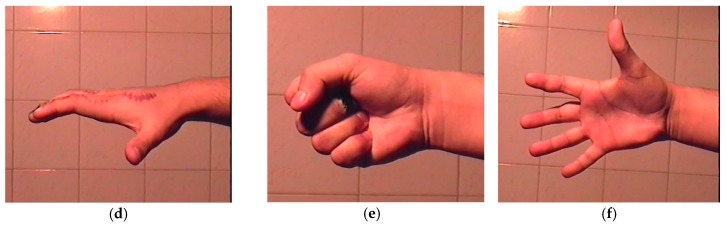
Clinical case of a repaired hand after a labour accident: (**a**) general view of PTN graft; (**b**) preoperative view of the injured hand; (**c**) postsurgical X-ray image, (**d–f**) range of hand motions in five days after surgery [107].

In the past three decades, PTN implants have been successfully deployed for surgical treatment of fractured bones since they showed remarkable efficiency [106]. A vivid example of the high biochemical and biomechanical compatibility of PTN is the use of cylindrical PTN grafts in hand surgery when repairing traumatic lesions and lost bone structures, as illustrated in Figure 15 [107]. Four damaged bone fragments resulted from a labor accident were substituted at once using cylindrical PTN grafts, which were customized intraoperatively.

The rough developed surface of PTN possesses self-adherence feature, whereas the porous structure maintains the ingrowth of host tissues herein. Due to the good rheological property and functional strength of the PTN graft, the range of hand motions was fully restored in five days after surgery. The patient was reported to continue his job three days after discharge. Follow-up observations evidenced the tight incorporation with host tissues and no complications. This highlights once more the functionality of PTN as a bone substitute when it uniformly redistributes high dynamic loads and, therefore, enables long-term cycling with no failure.

### 6.5. Cryotools Made of PTN

**Figure 16:**
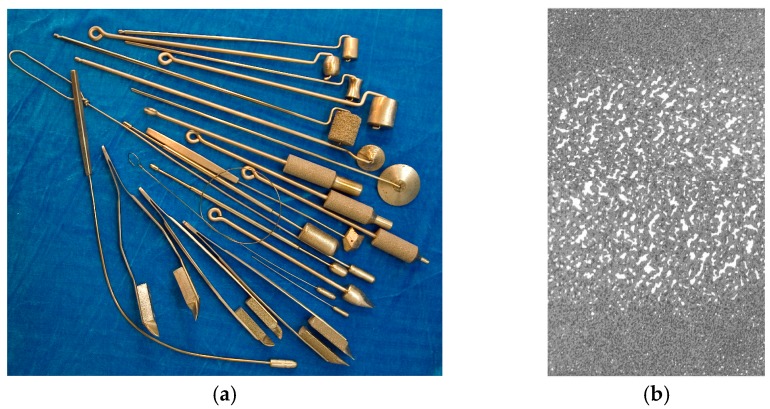
Images of (**a**) PTN cryotools and (**b**) variable porosity of the sectioned working part [64,108].

Cryotools having a working part made of PTN have been used in clinical practice for over 25 years due to the unique properties of the material [108]. Moreover, the flexibility of Nitinol rods/wires has allowed the fabrication of cryotools having the variable geometry handle seen in Figure 16, which is configured depending on application. From Figure 16, the working PTN part can be either unwrought or polished. Once the working PTN part has been immersed into liquid nitrogen, its changing color indicates how long the cryotool can be applied. Of course, the larger the working PTN part, the longer would be the cryoeffect. However, in a case of a minimally invasive approach or a hollow organ of smaller size, the appropriate cryotool needs to be chosen from [109,110]. Changing the SHS parameters, the inner structure of the PTN body was suggested to be intentionally designed to have a variable porosity in this regard (high-porous center and fine-porous periphery) [64]. This concept is feasible and helps to hold liquid nitrogen inside, preventing leakage as long as possible during cryo-application until the entire coolant content is evaporated.

**Figure 17:**
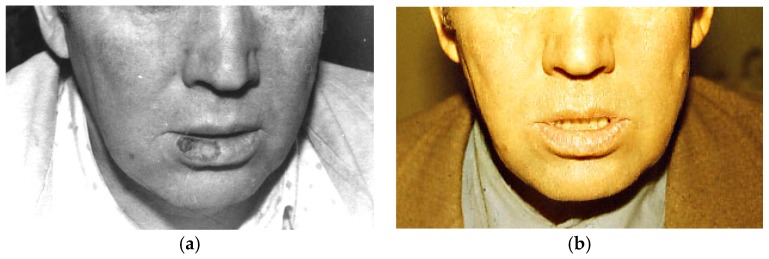
Images of a patient with precancerous orolabial lesion: (**a**) Before cryotreatment and (**b**) a month after treatment [104].

The variable and open-end porosity attains both a high permeability and low thermal conductivity of the working PTN part. In other words, the thermal screening effect ensures a lengthy cryo-exposure due to the higher performance, wherein up to 90% of the consumed coolant is transmitted to the contact surface. The main feature of PTN cryotools is that the working part does not adhere to applied tissues owing to the dry interface, which precludes ice formation. Such cryotools have been reported to be utilized in cryotherapy, cryosurgery, cryodestructive oncology, and skin care [104,108,111]. Figure 17 illustrates a clinical case for the cryotherapy of a precancerous orolabial lesion, where cryodestruction was performed using a cylindrical cryotool [108].

The biopsy probe has verified limited precancerous hyperkeratosis, whereas histological examination carried out six months after cryotreatment revealed a soft inconspicuous scar at the former affected area.

Finally, analysis of documented clinical cases using PTN devices over the past decade is given in Table 2. Spinal surgery has turned out to be the most sought-after field for implantable PTN devices, whereas cryotherapy has been in the forefront in the context of non-implantable devices.

## 7. Conclusions

The PTN biomaterial has been discussed in the light of material science engineering and more than three decades of clinical experience, and the following conclusions can be drawn:

(i) The biomechanical compatibility is referred to as the similarity of viscoelastic rheological characteristics between the PTN implant and host tissues. The combination of toughness inherent in Ti-based alloys, the porous morphology, and the viscoelastic reversible behavior of the porous body emphasizes the potential of PTN alloys to redistribute well physiological loads even in the early postoperative period, allowing to circumvent obstacles faced by existing implants;
(ii) The biochemical compatibility has turned out to be successful as well. The bioinertness of surface and inferential bioactivity evidenced through cytocompatibility and negligible foreign body reaction owe much to self-assembled superficial layers resulted from the SHS process and, as such, the as-received PTN implant does not require further surface modification;
(iii) Multifarious superficial layers demonstrating a complex structure/composition and high corrosion resistance conceal the matrix entirely and can be congruentially deformed without rupture and delamination, withstanding multicycle alternating loads;
(iv) The in vivo performance of PTN bone substitutes is also high. They may go through 10^7^ cycles with no failure due to the fact that chemical-proof layers arrest the surface deterioration, whereas the superelastic behavior of the matrix at alternating load rules out a possibility of the early material’s degradation.
(v) A large number of PTN devices have been clinically applied in traumatic/orthopedic surgery, maxillofacial surgery, spinal surgery, etc., due to a rare combination of structure, mechanical, and physicochemical properties of PTN as a biomaterial. Moreover, bioengineering can consider customized PTN grafts and PTN-based surgical techniques in the context of the next generation implants concerning surgery cost minimization and improved patient tolerance.
(vi) Comparative studies on corrosion fatigue behaviors of porous Ti and TiNi alloys made by both SHS and sintering are further needed to accomplish a complete and systematic understanding of PTN as an advanced biomaterial which can serve multiple clinical purposes.

## Figures and Tables

**Table 1:** Physical-mechanical properties of unwrought PTNs [3,12].

Property	Value
Specific weight, g/cm^3^	5.85
Porosity, %	60 to 75
Pore size, μm	0.1 to 200
Permeability coefficient (water/glycerin), m^2^	(0.27/62) × 10^−9^
Melting point, °C	1310
Ultimate tensile strength, MPa	100 to 500
Stretch at breaking point, %	5 to 7
Loading plato stress, MPa	50 to 200
Total elongation, %	2.5 to 4.5
Permanent set, %	5 to 20
SME recovery stress, MPa	200 to 400
SME temperature hysteresis, degree	30 to 100
Transformation temperature range, °C	−180 to 50

**Table 2:** Brief summary of clinically applied PTN devices from 2000 to 2010 [101,102,103,104,106,108].

Clinical Field	Embodiment of PTN	Number of Cases	Note
Traumatic and orthopedic surgery	Plates, Bars, Round bars, Stripes, Tapers, Customized endografts, Pellets	621	Open/closed bone fracture—361 Traumatic bone/joint lesion—127 Posttraumatic joint contracture—62 False joint—38 Congenital bone abnormality—23 Ankylosis—10
Spinal surgery	Customized cages (cylinder, bar, wedge etc.)	1983	Lumbar anterior/posterior interbody fusion: L_5_-S_1_—959; L_4-5_—791; L_3-4_—233
		641	Cervical anterior/posterior interbody fusion: C_2-4_—190; C_4-7_—451
		257	Spinal stenosis surgery: Lumbar—214; Cervical—43
Maxillofacial surgery	Plates, Bars, Round bars, Tapers, Stripes, Customized endografts, Pellets	409	Radicular cyst—175 Ameloblastoma—81 Odontoma—56 Osteoma—33 Condylar joint replacement—29 Total/subtotal mandibular replacement—25 Giant-cell tumor—10
Cryo-surgery/therapy	Cryotools having different size/shape/surface of the working part	1314	Cryodestruction of cutaneous and subcutaneous tumors (malignant, premalignant, non-malignant)
		200	Cryodestruction of hemorrhoidal boluses
		138	Cryotherapy of the obstructed urethra (urethral patency restoration)
		<9000	Cryotherapy of hemangioma in infants and pediatric patients
Oncosurgery	Plates, Disks, Round bars, Stripes, Customized endografts, Pellets	617	Bone/joint post-resection repair—322 Head and neck sparing surgery—295

## Supplementary Materials

The following are available online at https://www.mdpi.com/1996-1944/12/15/2405/s1. Video S1: Cervical spine dynamic PTN cage.
